# Cell-by-Cell: Unlocking Lung Cancer Pathogenesis

**DOI:** 10.3390/cancers14143424

**Published:** 2022-07-14

**Authors:** Ansam Sinjab, Zahraa Rahal, Humam Kadara

**Affiliations:** Department of Translational Molecular Pathology, The University of Texas MD Anderson Cancer Center, Houston, TX 77030, USA; zrahal@mdanderson.org (Z.R.); hkadara@mdanderson.org (H.K.)

**Keywords:** lung cancer, lung adenocarcinoma, single-cell sequencing, early detection, prevention, premalignancy, tumor heterogeneity, lineage plasticity, tumor microenvironment

## Abstract

**Simple Summary:**

Advances in lung cancer screening have led to a growing need for early intervention strategies for the expanding cohort of patients being diagnosed with the number one cancer killer. Such efforts heavily rely on an in-depth understanding of the pathogenic processes affecting normal lung epithelium and its surrounding microenvironment at the cellular level. More recently, advances in single-cell sequencing approaches have decoded much of the previously elusive complexity of lung malignant processes. Here, we review some of the major contributions of single-cell-based approaches that were employed to enhance our understanding of non-small cell lung cancers, and more specifically, lung adenocarcinomas. We focus on how studying lung adenocarcinomas at a cell-by-cell level continues to unravel an unbeknownst level of intratumor heterogeneity, identify plastic cell states at the heart of tumor inception, and elucidate the complex biology of premalignant progression, thus guiding novel approaches for clinical management of this deadly disease.

**Abstract:**

For lung cancers, cellular trajectories and fates are strongly pruned by cell intrinsic and extrinsic factors. Over the past couple of decades, the combination of comprehensive molecular and genomic approaches, as well as the use of relevant pre-clinical models, enhanced micro-dissection techniques, profiling of rare preneoplastic lesions and surrounding tissues, as well as multi-region tumor sequencing, have all provided in-depth insights into the early biology and evolution of lung cancers. The advent of single-cell sequencing technologies has revolutionized our ability to interrogate these same models, tissues, and cohorts at an unprecedented resolution. Single-cell tracking of lung cancer pathogenesis is now transforming our understanding of the roles and consequences of epithelial-microenvironmental cues and crosstalk during disease evolution. By focusing on non-small lung cancers, specifically lung adenocarcinoma subtype, this review aims to summarize our knowledge base of tumor cells-of-origin and tumor–immune dynamics that have been primarily fueled by single-cell analysis of lung adenocarcinoma specimens at various stages of disease pathogenesis and of relevant animal models. The review will provide an overview of how recent reports are rewriting the mechanistic details of lineage plasticity and intra-tumor heterogeneity at a magnified scale thanks to single-cell studies of early- to late-stage lung adenocarcinomas. Future advances in single-cell technologies, coupled with analysis of minute amounts of rare clinical tissues and novel animal models, are anticipated to help transform our understanding of how diverse micro-events elicit macro-scale consequences, and thus to significantly advance how basic genomic and molecular knowledge of lung cancer evolution can be translated into successful targets for early detection and prevention of this lethal disease.

## 1. Introduction

Lung cancer is the most commonly diagnosed malignancy worldwide [1]. With more than 1.8 million people dying of the disease annually, lung cancer accounts for the vast majority of cancer-related deaths worldwide and across both males and females [2]. The large mortality is, in part, due to late diagnosis after regional or distant spread of the disease [3,4]. Non-small cell lung cancer (NSCLC) is the lung cancer subtype that accounts for the majority of lung tumors (∼85%) [4]. NSCLC itself can be further divided into four subtypes, among which lung adenocarcinoma (LUAD) is the most prevalent subtype and the most common primary lung tumor overall [4,5].

Ever since the implementation of low-dose computed tomography (LDCT) chest screening among high-risk individuals with smoking history, lung cancer mortality rates have witnessed a steady decline [6]. With the increasing use of LDCT screening, more LUADs are being detected at early stages, thus requiring thorough evaluation and adequate treatment intervention. More recently, clinical management of LUAD has witnessed advances in therapeutic options whereby immune checkpoint blockade (ICB), which includes anti-PD-(L) 1 and anti-CTLA4-based therapies, has led to successful responses with durable efficacy including prolonging survival, thereby indelibly transforming the standard practice of LUAD treatment [7,8,9]. These advances have also inspired the interrogation of combinatorial neoadjuvant therapies (immunotherapy plus chemotherapy, or combinatorial ICB) in patients with resectable NSCLC [10,11]. Furthermore, and based on new and promising results from phase III CheckMate 816 trial, FDA approval was recently granted for the use of nivolumab combined with chemotherapy for resectable NSCLC, marking the first approval for neoadjuvant therapy in early-stage NSCLC [12].

Despite these advances, lung cancer continues to pose a significant public burden that is heightened by its dismal overall prognosis, poor clinical outcome, and a five-year survival rate of only 18% in the US, which is among the lowest across early-stage malignancies [13]. These sub-optimal outcomes are attributed to multiple factors, including low screening adherence (4–6% of potential eligible candidates in the United States) [14] as well as resistance of certain of patient subgroups to specific treatment strategies [15]. For instance, durable benefits in response to single-agent ICB are limited to a subset of patients, and this setback highlighted an urgent need to better identify biomarkers to stratify patients who are likely to respond to ICB and to determine the best window of time for therapeutic intervention [16]. Driven by this health conundrum and to develop effective strategies for early detection of lung cancer, much effort is being invested into understanding the events that promote tumor development and that would constitute ideal markers for early detection for the various subtypes of lung cancer, including LUAD.

Different NSCLC subtypes arise from distinct anatomical locations in the lung as well as from disparate cells of origin [17]. LUADs tend to develop from the lung periphery, whereby alveolar cells are suggested to be the LUAD cells-of-origin [5,18]. LUADs also exhibit a high degree of heterogeneity among patients and between cells of the same tumor tissue, and this has been shown in a number of studies largely focused early- to late-stage tumors and whereby cells were profiled by bulk approaches [19,20]. While several studies interrogating LUAD tissues have identified as possible markers (e.g., epithelial and immune) that promised strong clinical relevance for the growing subset of LUAD patients, a sobering corollary remains whereby these advances are of limited use in the context of predicting progression or clinical aggression of early stage lesions. Therefore, and to address this gap in knowledge, there has been a great interest in decoding the genomic, transcriptional, and molecular landscape of the earliest detectable stages preceding the development of aggressive lung cancers, including LUADs.

By taking a step back and interrogating lung premalignant lesions (PMLs), scientists were able to construct a timeline for the initiation and progression of certain lung cancer subtypes [17]. Despite improved efforts in clinical detection of lung cancers at early stages in patients, lung PMLs are increasingly difficult to distinguish, and resection of some premalignant subtypes is not the standard-of-care in many parts of the world [21]. Therefore, studying lung PMLs at high resolution and within large cohorts has been a bold and challenging quest in lung cancer research. As a surrogate, implementation of mouse models of lung cancer development has helped decode much of what we currently know of the landscape of early lung cancer pathogenesis [22]. More recently, the advent of single-cell sequencing technologies has uncovered new prospects for in-depth analysis of the transcriptomes, genomes, and proteomes of single cells. Single-cell sequencing also enabled the identification of rare cellular subsets which have been, for the most part, indiscriminable in bulk sequencing approaches where admixed populations of cells are analyzed, and underlying cell-specific differences are averaged out [23]. This review will focus on recent advances in dissecting LUADs at high resolution using single-cell-based approaches, and how these studies are providing novel insights into early stages of epithelial transformation. We will also highlight how this knowledge is painting a more complex portrait of lineage plasticity programs and intra-tumor heterogeneity which are prominent in the LUAD ecosystem. These advances bring forth valid reasons to interrogate the detailed spatiotemporal architecture of the lung’s earliest PMLs at the single cell level, a new and ambitious undertaking that holds great promise for LUAD research. Finally, this review will discuss the urgent need for a better understanding of LUAD premalignancy at high resolution, challenging areas to circumvent, and providing an optimistic outlook for the future of LUAD clinical management.

## 2. Epithelial Culprits of LUAD Transformation

### 2.1. LUAD Cell-of-Origin

Several studies have identified cellular epithelial candidates for transformation across multiple lung cancer subtypes, including LUADs. Newly discovered roles of specific epithelial subsets in LUAD oncogenesis are now being increasingly supported by single-cell sequencing and lineage tracing studies using genetically engineered mouse models (GEMMs) [24]. Historically, LUAD initiation has been shown to be driven by a combination of environmental (e.g., inflammation that enhances the transformation of cells with specific genotypes [25]) and genetic factors, with the latter showing variable roles depending on the specific epithelial lineage where the driver mutation is expressed. One of the most frequently studied mouse models of lung cancer LUADs driven by *Kras* mutations. Mutations in the *Kras* oncogene constitute the most common oncogene-driven subtype of LUAD, with multiple reports showing that these mutations constitute a major event in early LUAD tumorigenesis. As such, researchers have utilized a variety of approaches to interrogate mouse models with conditional spatial and temporal expression of mutant *Kras*^G12D^; these GEMMs express the mutant *Kras* allele at specific timepoints and/or across defined mouse epithelial compartments with the aim of identifying LUAD cells-of-origin. Based on a number of published studies in the murine lung, *Kras*-mutant LUAD preferentially arises from alveolar type 2 (AT2) cells [25]. In agreement with these findings, alveolar staining marks LUADs diagnosed at early stages, and generally LUADs are most frequently diagnosed at the distal airway where AT2 cells are found [25,26,27,28]. In contrast, transformation of club cells by targeting *CC10* promoter expression does not lead to detectable LUAD formation despite the presence of hyperplasias at the bronchoalveolar duct junction [26,27].

### 2.2. Single-Cell Sequencing Studies Open a Pandora’s Box of Pulmonary Cellular Heterogeneity

Despite this knowledge, the full picture of how transformation of AT2 cells leads to development of PMLs and invasive LUAD is far from complete. This notion becomes more convincing with recent studies showing that the hierarchies, functions, and states of normal lung cells in both human and mouse are far more complex than we had previously thought. Indeed, genome-wide expression profiling of lung epithelial cells by single-cell sequencing technologies culminated the apex of these endeavors. Thus, it not surprising to see that decoding the roles of individual cellular subsets in lung cancer pathogenesis, including AT2 cells, lung cancer cell-of-origin, and beyond, has greatly benefited from studies focused on charting a detailed landscape of the lung epithelial lineages and their states during normal lung development and homeostatic control (e.g., in response to acute stress signals). For example, Angelidis et al. noted that the ageing lung exhibited deregulation of epigenetic control and subsequently increased transcriptional noise [29]. Their in-depth single-cell analyses enabled them to pinpoint significant changes in the relative frequencies of airway epithelial cells and to identify unique states within specific lung subsets that are implicated in the ageing process, such as increased cholesterol biosynthesis in AT2 epithelial cells and lipofibroblasts. By charting a single-cell atlas of the normal lung, Travalgini et al. identified 14 new cell types, including a comprehensive mapping of the transcription factors and markers of lung cells, and subsequently, their biochemical functions, cellular crosstalk, developmental roles, and possibly their deregulation in respiratory illnesses [30]. By analyzing and comparing murine and lung single-cell lung atlases, the authors pinpointed expression switches between cell types, thereby pointing towards a high level of lineage plasticity during mammalian lung organ evolution [30]. Single-cell analysis of lineage hierarchies and mapping of unique alveolar developmental states in the mouse lung led Treutlein et al. to the identification of bipotential alveolar progenitors [31]. Similarly, single-cell RNA-sequencing (scRNA-seq) analysis aided the identification of a subpopulation of alveolar cells with deregulated cellular metabolism and that was also implicated in the pathobiology of advanced chronic obstructive pulmonary disease (COPD) [32]. Plasschaert and colleagues identified a rare and previously unknown lung epithelial cell by scRNA-seq of proximal airway epithelium during homeostasis and regeneration [33]. Coined as the “pulmonary ionocytes”, the authors showed that these cells are a major source of CFTR activity, a finding that holds great promise for future therapies for cystic fibrosis [33]. Furthermore, a number of single-cell studies consistently identified epithelial and mesenchymal lineages whose unique transcriptional states and spatial localization are highly relevant to the pathobiology of pulmonary fibrosis [34,35,36]. In all these studies, the implementation of scRNA-seq technologies constituted a major pillar in providing detailed knowledge of rare or transitional subsets as well as cell-type-specific mechanisms at unprecedented depths.

Given the many parallels between the pathobiology of cancer and that of some of these aforementioned diseases (e.g., COPD, fibrosis), or with unique transcriptional states (e.g., stem cell-like potential, inflammatory responses), it is not unlikely that some of these single-cell mechanisms described in normal lung development, regeneration, and homeostatic regulation are also implicated and/or deregulated in lung cancer pathogenesis. In fact, the recent burst and ongoing expansion of in-depth analyses of the transcriptomic architecture of lung epithelial, immune, and stromal subsets at the single-cell level has spearheaded several discoveries that extend beyond identifying new cell subsets and states in the lung. In the next section, we will summarize a number of single-cell-based studies investigating tumorigenic lung tissues, particularly LUAD, and how these interrogations enabled a better understanding of the LUAD and its tumor microenvironment (TME), heterogeneity (in cellular composition, pathobiology, genomic variability in mutations, single-nucleotide and copy number variants, chromatin accessibility, and gene expression signatures), as well as developmental trajectories, cancer evolution, and premalignancy.

## 3. LUAD Epithelial Plasticity: In Times of Stress, Lineage Commitment Is a Rare Feat

By providing a more detailed account of the natural history of lung tumor cells and the molecular and cellular underpinnings of how these aggressive cancers evolve with time, single-cell studies are providing in-depth knowledge into the tumor cells-of-origin, role of tumor–immune interactions, niche factors that can modulate epithelial lineage plasticity and the susceptibility to form tumors, and the facets of heterogeneity within and across tumors. We are now beginning to understand how these factors impinge on tumor progression by studying the co-evolution of tumors and their ecosystem at the single-cell level, starting with early acquisition of oncogenic mutations (by comparing normal and malignant cells), to establishment of distant metastases (primary vs. metastatic tissues), and up to eliciting variable responses to therapeutic intervention (responsive vs. resistant tumors).

The histopathology of NSCLCs is not fixed over time, and this can be due to the deviation of tumor-initiating populations from their original lineages by dedifferentiating or transdifferentiating [37]. A prominent example of a developmental process with clear manifestation of epithelial plasticity is epithelial-to-mesenchymal transition, a process that is highly pertinent for multiple stages of tumor development including early stages of initiation [37]. With lineage plasticity manifesting as a pervasive phenomenon in the evolution of multiple tumors, it is not surprising to see that it is now considered one of the new hallmarks of cancer [38]. Specifically for LUADs, a gene module of normal alveolar differentiation stratifies tumors with distinct grades, biological properties, and clinical outcome [39]. Studying LUAD pathogenesis at the single-cell level is elucidating a growing role for aberrant cellular plasticity programs (e.g., in alveolar subsets) in space or time, throughout LUAD inception and evolution, as summarized in this section and Table 1. In accordance with the role of alveolar subsets in early LUAD pathogenesis, Wang et al. detected, by scRNA-seq, a subset of AT2-like cells that emerged in AAHs and that became more transcriptomically divergent from normal AT2 cells as the LUADs progressed [40]. This AT2 subset showed early activation of energy metabolism and ribosome synthesis programs, and later adopted stem-cell like properties, thereby pointing to early epithelial states that are implicated in LUAD pathogenesis [40]. Phenotypic plasticity in AT2 cells has been also identified by Wu and colleagues whereby single-cell analysis of epithelial subsets of advanced stage LUADs identified two major clusters of AT2 cells, one of which expressed malignancy marker genes and that could transition to tumors as shown by inferred trajectory analysis [41]. Recent evidence from continuous, high-resolution in vivo tracking of cancer cells in lineage tracing mice with oncogenic *Kras* and *p53* mutations enabled the identification of divergent cellular plasticity and evolutionary paths, distinct expression programs that expand during tumor evolution, and the formation of metastases from spatially localized, expanding subclones of the tumor [42]. In a large-scale analysis of 30 patients with late-stage NSCLCs before and during targeted therapy, Maynard et al. identified that malignant cells harbored potentially targetable oncogenes that are beyond those detected clinically, and those that survived targeted treatment exhibited lineage plasticity and adopted an alveolar-regenerative cell signature, suggestive of a primitive transitionary cell state that is triggered by therapy [43]. Laughney and colleagues showed that in primary LUADs, epithelial cells adopt regenerative states resembling normal cellular responses to injury, and that almost all alveolar and bronchial lineages exhibit a high level of promiscuity [44]. Single-cell transcriptional branches showing deviation from normal ciliated or alveolar differentiation programs were also shown in a systemic single-cell analysis of metastatic LUADs [45].

Multiple lines of evidence have previously shown that changes in molecular or extracellular signals can directly tamper with phenotypic plasticity, for instance, by restricting the expression of transcription factors and cofactors to specific cell lineages [46,47]. Penkala et al. described a mechanism whereby plasticity contributes to lung regeneration during neonatal life, along the AT1-to-AT2 pathway [48]. In the adult lung, AT2 cells are known to possess regenerative properties that aid in maintaining tissue homeostasis. In fact, transitional alveolar states along the AT2-to-AT1 differentiation pathway have been recently detected by scRNA-seq approaches and in response to insults to mouse lung tissues (e.g., bleomycin or hyperoxia) [49,50,51]. Overall, these cells show increased DNA damage and can be caught up and increase in number in cases where the damage is not cleared, thereby leading to homeostatic imbalance and diseased lung states such as fibrosis. Therefore, it is tempting to suppose that in the presence of tissue damaging and tumor-promoting insults (e.g., carcinogenic, chronic inflammation), emerging intermediary cell states could also be the missing link along the transition of normal alveolar cells to premalignancy [49]. This conjecture has been supported by the existence of these intermediary cell states in human LUAD tissues [52,53], but more work is needed to pinpoint the precise extracellular triggers and to map the subsequent cellular transitions at the singe-cell level, and how these factors collectively contribute to lung pathogenesis.

## 4. Intratumor Heterogeneity at the Single-Cell Level: One Size Does Not Fit All

As more reports investigating lung cancers at the single-cell level emerged, an unprecedented level of variability within a single tumor, or intra-tumor heterogeneity (ITH), was brought to light. For decades, tumor morphological heterogeneity has been documented by pathologists. The advent of deep sequencing technologies made it further possible to pinpoint causes of ITH, including genetic and epigenetic variability, interactions of tumor cells with surrounding cells, proximity to blood vessels, and presence of tumor-infiltrating lymphocytes. While the majority of these studies were based on a deep sequencing approach to analyzing admixed populations of cells within a single tumor site, micro-dissected tissues, or clonal outgrowths, the findings provided a much more complex view of tumor evolution and clonal dynamics, as well as clinical implications of ITH, including prognosis, response to therapy, and relapse. For instance, independent groups conducting multiregional sequencing of LUADs revealed a high degree of ITH between different sites within the same tumor which, upon further investigation, revealed a branched evolutionary trajectory across the majority of LUADs [54], as described further below.

Multiregional sequencing has been the most successful strategy to investigate intratumor heterogeneity and clonal evolution in LUAD to date [55,56,57]. These studies provided deep insight into the early role of alterations in canonical driver genes, chromosomal instability in generating extensive subclonal divergence at later stages of tumor evolution, and the role of non-canonical cancer genes in driving tumor development and subclonal diversification. The results also highlighted diverse associations between the genomic landscape of lung tumors and clinicopathological features as well as response to therapy. The findings also beg the burning questions of how can we standardize sampling to confidently assess the extent of ITH in patients: if single-region sampling is insufficient, how many multi-region samples would be representative of the whole tumor? What is the minimal sequencing depth required to detect driver and subclonal mutations, including those that are found in rare but tumor-pertinent subsets of epithelial cells?

**Figure 1:**
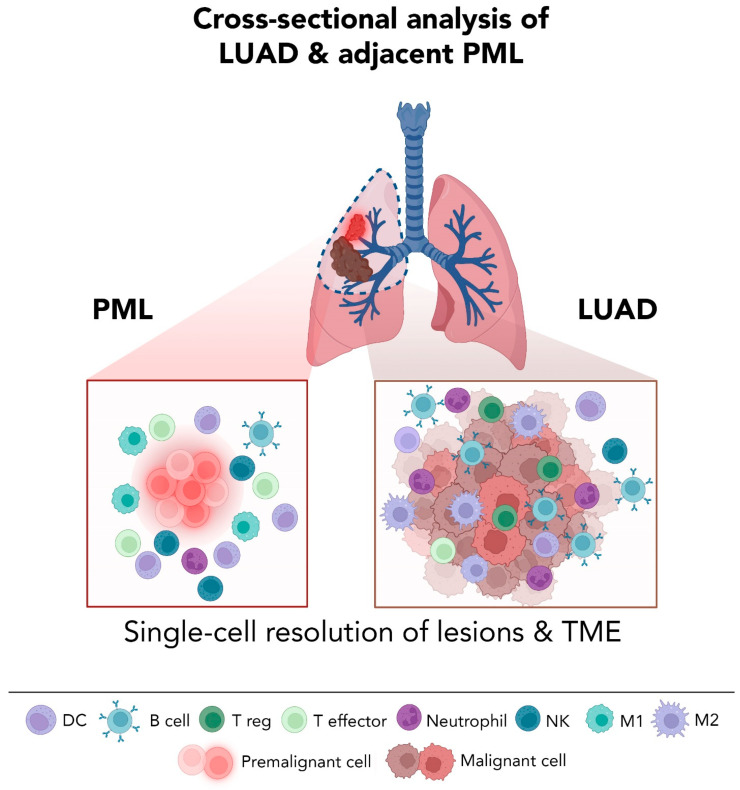
A high-resolution overview of lesions and the TME revealed by cross-sectional single-cell analysis of LUADs and adjacent PMLs found in surgical samples from patients (e.g., lobectomies). Analysis of lesions at single-cell resolution can reveal in-depth insights into the biology of epithelial and non-epithelial (e.g., immune) subsets. This can help delineate the dynamics of epithelial plasticity and intra-tumor heterogeneity and point towards possible ways to intercept and impede the development of PMLs and/or their progression to LUADs. PML; premalignant lesion. LUAD; lung adenocarcinoma. DC; dendritic cell. Treg; regulatory T cell. NK; natural killer cell. M1; M1 type macrophage. M2; M2 type macrophage. Created with BioRender.com.

Many of these hurdles can now be circumvented by adopting single-cell approaches which offer a new level of granularity to decode LUAD heterogeneity, to understand the consequences of ITH with respect to tumor pathobiology, and to potentially predict LUAD response to therapy (Figure 1 and Table 1). For example, by applying scRNA-seq to analyze enriched epithelial subsets (by flow cytometry) from early-stage LUADs and multi-region samples of the tumor niche (normal-appearing lung tissues at different spatial distances from the tumors), our group was able to interrogate the continuum of topographically spatial normal appearing tissues representing a “field” that has been only studied previously by bulk sequencing approaches [58]. The results pointed towards a high degree of heterogeneity in epithelial lineage plasticity (e.g., alveolar subsets) and distinct tumor cells of origin [53,58]. In fact, one can think of studying the “cancerized field” of normal-appearing tissues in the vicinity of tumors as a surrogate for the identification of premalignant events in early tumor development, and interrogating these alterations at the single-cell level may help derive targets for early interception [59] (Figure 1).

Single-cell transcriptomic resolution is also uncovering ITH in LUAD immune and stromal tumor compartments, in addition to epithelial cells. This is not surprising, since it is now well known that the dynamic interplay between cancer cells and the TME plays key roles in cancer pathogenesis [60,61]. For instance, failure of deployment of cytotoxic T-cell lymphocytes (CTLs) that can target tumor cells or suppression of immune surveillance and effector (anti-tumor) responses by immune inhibitory checkpoints is thought to play key roles in cancer progression [60,61,62], including in LUAD [63]. Additionally, increased levels of tumor infiltrating CTLs are a favorable prognostic indicator of LUADs [64]. Generally speaking, and relative to normal tissues, lung tumors are enriched with M2 macrophages, plasmacytoid dendritic cells (pDCs), regulatory T cells (Tregs), exhausted CD8^+^ T cells, B cells, and particularly plasma cells, whereas they are devoid of natural killer (NK) cells and blast B cells [65]. Despite these established insights, a decent number of single-cell interrogations uncovered a much more complex epithelial, immune, and stromal landscape of NSCLCs and LUADs and unearthed deeper layers of ITH at the level of transcriptional states, as summarized henceforth and in Figure 1.

In our single-cell analysis of non-epithelial subsets along the spatial continuum of normal-appearing lung tissues (tumor-distant to -adjacent) and matching early-stage LUADs (see previous paragraph), we identified changes in immune subsets that evolved with increasing proximity to the tumor and that could signify early alterations in the TME, such as increased signatures and fractions of Tregs, as well as reduced cytotoxic CD8^+^ T cells, antigen-presenting macrophages, and inflammatory DCs [58]. By analyzing LUADs spanning the normal-to-metastatic pathogenic continuum, Kim et al. showed that tumors elicit early stromal and immune alterations that provoke a pro-tumoral and immunosuppressive TME and that are sustained until later stages [45]. This single-cell report specifically highlighted how monocyte-derived macrophages and DCs, as well as exhausted T-cells, gradually outstrip normal resident myeloid populations [45].

In one study focused on scRNA-seq analysis of tumor infiltrating myeloid cells (TIM), researchers identified an overlap between murine and human TIM subsets in NSCLCs [66]. The analysis also identified transcriptomically distinct human TIM subsets, including one DC and two neutrophil and subsets that were associated with improved or poor patient survival, respectively. Overall, the results provided a comprehensive catalogue of the different myeloid populations in the malignant lung, and an introduction to additional studies interrogating the clinical implications myeloid ITH [66]. The functions of tumor associated neutrophils in NSCLC was further shown to be associated with tumor heterogeneity, which was found to be highly prevalent in NSCLCs analyzed at the single-cell level [41]. Non-canonical roles for tumor-resident neutrophil subsets was also recently reported by Salcher and colleagues, who were able to sequence technically challenging subset of cells with low mRNA content [67]. The authors derived a neutrophil signature that identified patients who are refractory to treatment with PD-L1 inhibitors [67].

In a multimodal approach, one group coupled cell-level transcriptomes (by scRNA-seq) and surface epitope expression (by Cite-Seq) derived from the same cells and across 35 NSCLCs [68]. The report identified a lung cancer activation module (LCAM) that was strongly related to tumor mutational burden, ectopic antigens, and driver mutations [68]. The large-scale analysis further enabled the authors to identify a prognostic benefit for LCAM in patients receiving anti-PD-L1 immunotherapy [68].

T-cell-centered scRNA-seq analysis of 14 NSCLC patient samples revealed that NSCLCs enriched with a CD8^+^ T cell pre-exhausted subset had better prognosis [69]. A subset of CD8^+^ terminally differentiated effector memory or effector cells was also found to be increased in some NSCLCs by scRNA-seq analysis, pointing toward high degree of intra-tumor heterogeneity in the TME [70]. In this report, the authors derived T cell related signatures that correlated with improved survival of LUAD patients [70]. Lambrechts et al. took a slightly different direction whereby single-cell annotation of the transcriptome of lung tumors, mostly LUADs, was focused on TME and stromal populations [71]. Their analysis identified 52 distinct stromal subtypes (e.g., endothelial cells, cancer-associated fibroblasts, and TILs) [71]. By analyzing their abundance, the authors identified correlations between some of these subsets and patient survival and/or tumor stage. For instance, an exhausted subset of CD8^+^ cytotoxic T cells with high expression of *LAG3* was inversely correlated with survival [71]. Similarly, Bischoff and colleagues unveiled a high degree of LUAD heterogeneity at the single-cell level which represented distinct tumor histologies and variable activation of oncogenic pathways [72]. Analysis also showed that LUADs adopt one of two distinct TME patterns: a prognostically unfavorable pattern with high prevalence of cancer-associated myofibroblasts, proinflammatory monocyte-derived macrophages, pDCs, and exhausted CD8^+^ T cells, in contrast to a prognostically favorable and inert TME characterized by normal-like myofibroblasts, non-inflammatory monocyte-derived macrophages, NK cells, myeloid dendritic cells, and conventional T cells [72]. scRNA-seq of the TME of NSCLCs can be also employed to decode possible mechanisms of response to ICB. In one report analyzing 36 patients, a unique subset of precursor exhausted CD8^+^ T cells with low expression of coinhibitory molecules and high *GZMK* was found to significantly associate with tumors that were responsive to anti-PD-1 treatment [73]. In the study of Maynard and colleagues (described above), a high degree of heterogeneity in cancer cells and the TME within and across tumors was identified [43]. In addition to highlighting lineage plasticity and heterogeneity at high resolution, single-cell studies can also shed light on mechanisms by which the TME can directly shape tumor progression. For instance, single cell analysis of metastatic LUADs identified cell states that span the continuum of metastatic progression; the spectrum showed overall reduced cellular differentiation and more prominent stem cell-like properties, as well as evidence that developmental plasticity can be directly sculpted by NK cells [44].

Some attention has been also directed towards B cell heterogeneity in NSCLCs at the single-cell level. For instance, distinct infiltrating B cell subtypes identified by scRNA-seq were linked to NSCLC progression and clinical implications in a context-specific manner [74]. The authors reported that low levels of naïve-like B cells and an association with poor prognosis were prominent in advanced NSCLCs [74]. In contrast, plasma-like B cells exhibited anti-tumor and pro-tumor growth functions in early vs. advanced stage NSCLCs [74].

The findings from these single-cell approaches to decode NSCLCs and particularly LUADs uncovered novel players in tumor development, progression, and/or response to therapy that contribute to a high level of ITH, which has been, for the most part, elusive (Table 1). By extending knowledge harnessed from scRNA-seq approaches to studying larger and more diverse cohorts at higher depth, we can begin to better understand epithelial-TME crosstalk at early stages of tumor inception, and thus derive targets for interception and prevention, as described further below.

**Table 1:** List of LUAD scRNA-seq studies that are summarized in this review and grouped by LUAD phenotypes and features.

LUAD Phenotypes and Features	Disease Stage/Model	Major Discoveries	Ref.
Lineage commitment, plasticity	AAHs, LUADs	AT2 lineage-divergent subset appears in AAHs and up to LUAD development, activated metabolic and stem-cell-like programs.	[40]
	Advanced stage LUADs	AT2 subset with malignant properties and potential to transition to tumor cells.	[41]
	In vivo tracing in *Kras*; *Trp53*-driven mouse model of LUAD	Clonal evolution with divergent lineage plasticity programs.	[42]
	Metastatic LUADs	Altered ciliated and alveolar differentiation programs.	[45]
Alveolar intermediary/ regenerative states	Late-stage LUADs in response to targeted therapy	New potentially targetable oncogenes. Residual malignant cells are in a primitive regenerative alveolar state.	[43]
	LUADs	Alveolar and bronchial regenerative state mimicking response to injury.	[44]
Intratumor heterogeneity (ITH) in epithelial, immune, or stromal subsets, and relevance to clinical outcomes and therapy response	Enriched epithelial subsets from LUADs and multiple spatially defined normal tissues	ITH in epithelial lineage plasticity and distinct tumor cells of origin, and ITH in TME that evolved with increasing tumor proximity.	[53,58]
	Normal lung, LUADs, and metastases	Early pro-tumoral and immunosuppressive TME alterations (stromal and immune) are sustained until later stages.	[45]
	Tumor infiltrating myeloid cells in NSCLCs	Comprehensive catalogue of distinct myeloid populations (e.g., neutrophil and DC subsets) linked to survival outcomes.	[66]
	NSCLCs including LUADs	ITH is highly linked to tumor-resident neutrophil subpopulations.	[41]
	scRNA-seq of low mRNA neutrophil subsets in NSCLCs	Neutrophil signature associated with poor response to ICB.	[67]
	scRNA-seq and Cite-Seq in NSCLCs	A prognostically-relevant module for ICB patients based on tumor mutational burden, ectopic antigens, and driver mutations.	[68]
	NSCLCs including LUADs	CD8^+^ T cell pre-exhausted subset associated with better prognosis.	[69]
	NSCLCs including LUADs	Increased CD8^+^ terminally differentiated effector memory or effector cells in tumors. T cell signatures correlated with improved survival.	[70]
	LUADs	52 distinct stromal subtypes, some correlated to patient survival and/or tumor stage.	[71]
	LUADs	ITH in tumor histologies, oncogene pathways, and TME patterns signifying distinct prognostic outcomes.	[72]
	NSCLCs including LUADs receiving ICB	Responsive tumors associated with precursor exhausted CD8^+^ T cells with low expression of coinhibitory molecules and high *GZMK.*	[73]
	Late-stage LUAD response to targeted therapy	TME heterogeneity within and across tumors.	[43]
	Metastatic LUADs	NK cells sculpt developmental and epithelial plasticity throughout metastatic progression.	[44]
	Early and advanced stage NSCLCs including LUADs	ITH resulting from distinct B cell subtypes (e.g., naïve- or plasma-like B cells) linked to progression and clinical implications in early or late stages.	[74]

AAH; atypical adenomatous hyperplasias, LUAD; lung adenocarcinoma, AT2; alveolar type 2 cells, NSCLC; non-small cell lung cancer, ITH; intratumor heterogeneity, TME; tumor microenvironment, DC; dendritic cell, ICB; immune checkpoint blockade, NK; natural killer.

## 5. The Urgency in Premalignancy

**Figure 2:**
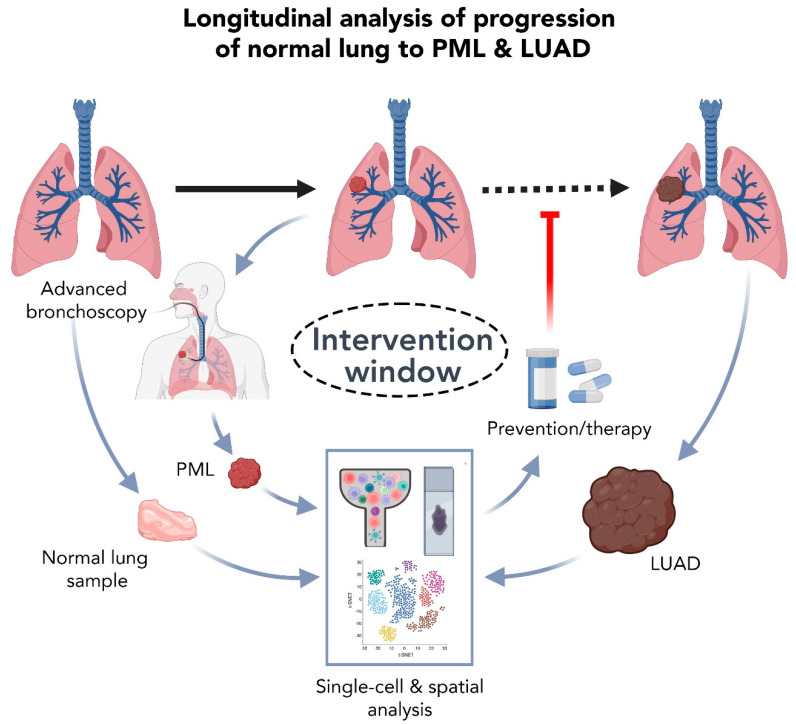
Longitudinal single-cell analysis of normal lung tissues, PMLs, and LUADs underscores new challenges and offers a unique window of opportunity for early prevention and treatment. Advances in bronchoscopic isolation of PMLs can potentiate routine single-cell and spatial analysis of early lung lesions, matching normal lung tissues and LUADs along the pathogenic continuum of disease progression. By comparing malignant cells and components of the TME at the single-cell level, we can begin to tease out cell-level intrinsic and extrinsic changes, states, or mechanisms that can be targeted at early stages to prevent initiation and progression of lung lesions. Analysis of additional samples such as late-stage (e.g., metastatic) lesions or tissues from patients before vs. after treatment can also help identify, at high resolution, factors that enable tumor dissemination and modulate response to therapy, respectively. Such analyses can thus help improve pathological annotation, therapeutic stratification, and clinical outcomes for LUAD patients. PML; premalignant lesions, LUAD; lung adenocarcinoma. Created with BioRender.com.

While high resolution mapping of lung cancer tissues can provide novel insights into the biology of these diverse tumors, the best window for promising clinical intervention remains early along the progression of lung cancer (Figure 2). The majority (~85%) of lung cancer cases are diagnosed in lifetime (current and former) smokers and exhibit poor prognosis and inferior response to systemic or targeted therapy [75,76]. However, despite the causal effect of smoking on lung cancer development, only 15% of smokers develop lung malignancy in their lifetime [3,4,75,76]. Healthy patients may have subclinical disease for years prior to presentation and diagnosis, but without the ability to detect and characterize PMLs, strategies that prevent or treat lung cancer in its earliest stages in high-risk individuals (such as smokers) remain far-fetched. The clinical challenge is to develop non-invasive lung cancer detection methods that are effective during this window of opportunity (Figure 2). This has guided several efforts to improve detection of lung PMLs and conduct cross-sectional and longitudinal analysis of tissues (Figure 1 and Figure 2) in order to dissect the earliest molecular transformations along the continuum of lung pathogenesis and thus identify pertinent targets for early detection, prevention, and/or treatment. In this section, we will summarize a handful of high-resolution analyses into the earliest known lesions of LUADs and provide an outlook on how advances in detection and sampling of this lesions, along with high resolution single-cell and spatial profiling, can broaden our horizons in early clinical management of lung cancer.

### 5.1. LUAD’s Earliest Known Preneoplastic Lesions

Cellular, molecular, and genetic anomalies have been described specifically for each lung cancer subtype, including oncogenic activation and prognostic as well as targetable mutations. A number of those alterations have been mapped along the continuum of lung carcinogenesis, including in premalignant phases of tobacco carcinogen-mediated lung cancers [77,78,79,80]. This enabled the identification of genetically, epigenetically, and molecularly diverse sequential phases of tumor progression for some subtypes of lung cancer, which may be explained by the divergent anatomical locations and cells of origin of the lung tumors, as previously described above. As such, our current lung cancer classification system now extends beyond late-stage tumors themselves, to deconstruct the phases preceding the transition into most of the known lung cancer subtypes. These phases describe the precursor or preneoplastic lesions of the bronchial epithelium, which are classified intro three different lesions according to the World Health Organization (WHO) [81,82]. For LUSCs, hyperplasia, squamous metaplasia, squamous dysplasia of increasing severity, and carcinoma in situ have been described to progress in a sequential manner [83]. For other lung tumor subtypes, fewer distinct and sequentially occurring precursor phases have been described. In contrast to other NSCLC subtypes, LUAD PMLs are less elusive. To date, the only known progenitor lesions for LUADs are atypical adenomatous hyperplasias (AAH) [5], which present as localized proliferation of AT2 cells measuring <5 cm and are found adjacent to 5–20% of resected LUADs, sharing genetic and epigenetic alterations with respective LUADs [84] (Figure 1). Adenocarcinoma in situ (AIS) and minimally invasive adenocarcinomas (MIA) were also suggested as two additional intermediate entities along the linear progression from pre-invasive lesions to LUADs, whereby AIS describes neoplastic cells growing adjacent to alveolar structures and measuring < 3 cm [81]. MIAs demonstrate a solid component by CT and are characterized by a focus of invasion measuring < 0.5 cm [81]. AAH and AIS appear as ground glass opacities or nodules (GGNs) on CT imaging, while MIAs demonstrate a solid component. These lesions likely constitute a continuum along which pre-invasive lesions progress to LUAD. In contrast, pre-neoplastic lesions of SCLCs remain, thus far, largely elusive, possibly because SCLCs do not progress in the same orderly manner seen for other lung cancer subtypes. In fact, normal-appearing mucosa carrying significant genetic abnormalities is often found near small cell tumors, suggesting that these tumors may progress without progressing through an intermediate stage of morphologically recognizable preinvasive lesion [85].

### 5.2. High Resolution Analysis of Premalignant LUAD Lesions: Promises and Challenges

Interrogation of premalignant tissues is a highly informative tool that bears strong clinical relevance for guiding treatment strategies. As outlined by Wilson and Junger’s principles for early screening, a recognizable latent or pre-clinical stage of disease is crucial for a successful implementation of cancer screening approaches, and this has been evident in cervical and colon cancer, which are characterized by a well-defined period of asymptomatic pre-clinical disease [86]. While this is highly relevant for lung cancer, whereby only 15% of smokers develop lung malignancy in their lifetime despite the causal effect of smoking on lung tumorigenesis [3,4,75,76,87], screening strategies for premalignant lung nodules remain riddled with obstacles. Low-dose CT (LDCT) imaging, now endorsed as an annual screening procedure for those at high risk for lung cancer, was shown to reduce mortality by 20% as reported by the National Lung Screening Trial [88,89]. However, LDCT screening is still contended with producing high false-positive screen rates, risking cumulative radiation exposure, and accounting for substantial economic costs [90]. Additionally, endobronchoscopy is often non-diagnostic in smokers with nodules, and prior to the recently growing implementation of advanced bronchoscopic navigational techniques (see section below), the use of conventional bronchoscopy to target, excise, and assess peripheral LUADs ex vivo has remained largely unattainable [90]. In addition, several lines of evidence point towards a non-uniform, highly heterogeneous lung cancer biology, and not all early lesions lead to invasive cancer [91,92]. Therefore, identification of premalignant specimens is key to advancing research aimed at understanding the biology of malignant progression, and subsequently, improving clinical diagnostics.

Establishing pre-cancer tissue cohorts is crucial for the interrogation of the earliest events that drive lung cancer oncogenesis, as well as for target validation. This could be significantly accelerated by the feasibility of studying human-derived specimens ex vivo. It is noteworthy to mention that understanding premalignancy entails not just decoding early epithelial transformation events but also mapping the precise changes in host immune programs, such as likely shifts from lung tumor immune surveillance to escape, that occur during primitive phases (e.g., progression from normal to premalignant states), and identifying these changes may help devise strategies for early immune-based interception and/or treatment (Figure 1). Such endeavors have been largely spearheaded by analyzing early LUAD lesions by single-cell sequencing approaches. Several studies thus went on to interrogate, by scRNA-seq, ground-glass nodules (GGNs), which account for most lung cancers detected in outpatient screening detected by LDCT and comprise an ideal model to study early forms of LUAD. Analysis of 11 GGNs and normal lung tissues from 6 never-smoker patients identified that the tumor-evasive TME is governed by Treg enrichment, and is characterized by early depletion of γδT and NK cells and infiltration of B cells and abundance of cancer-associated fibroblasts with unique gene expression profiles [93]. Furthermore, analysis of malignant subsets identified early loss of surfactant homeostasis, which could be implicated in early disease pathogenesis [93]. In one study [94], researchers compared the GGNs with solid adenocarcinoma (SADC) by scRNA-seq. Analysis identified that GGN cancer cells exhibited downregulation of signaling pathways related to cell proliferation and angiogenesis, distinct stromal cell transcriptomic states including reduced expression of collagens in fibroblasts, and increased activation of immune cells, relative to SADC cells. Another study interrogated, by scRNA-seq, the microenvironment of subsolid nodules (SSN) which often represent premalignancy (AAH, AIS, MIA) or early spectrum and indolent LUAD [84], and compared those to solid or uninvolved lung tissues from surgical resection lung specimens [95]. Analysis identified that both cancer-associated AT2 cells and fibroblasts contribute to the deregulation of the extracellular matrix, potentially affecting immune infiltration in subsolid lesions through ligand-receptor interactions. The authors also analyzed immune cells at single-cell level and found a significant decrease in the cytolytic activities of NK and NKT cells, accompanied by a reduction of effector T cells as well as an increased CD4^+^ regulatory T cells in subsolid lesions. In contrast, others have shown that the TME of SSN is dominated by cytotoxic NK/T cells, whereas metabolic reprogramming and immune stress responses were significantly observed in SSN malignant cells [96]. By comparing scRNA-seq data to normal and/or metastatic lung tissues, the authors also showed that the subtype composition of endothelial cells resembled that of metastatic LUAD, while the fibroblasts was more similar to those observed in normal lung [96]. Despite these insights, data from these studies ought to be interpreted with caution due to the elusive nature of these types of lung nodules. The reality is that in lung cancer, where surgery is not the standard of care for the clinical management of lung nodules, a comprehensive understanding of the molecular and genomic pathology of lung preneoplastic, preinvasive, and early invasive lung lesions remains largely hindered by the difficulty in identifying those lesions, the scarcity of resected specimens needed for currently available scRNA-seq platforms, and the low cellular fractions of somatic mutational processes detected within these premalignant tissues using bulk approaches.

A handful of research groups are now starting to integrate single-cell analysis of lung tissues (healthy or diseased) with unbiased high-resolution spatial mapping of cell types in situ. This provides a deeper understanding of the roles of newly discovered cell types, states, or transitions, as well as their inferred interactions with other cell subsets (e.g., immune or stromal) in the geospatial context of the surrounding TME. In cancer tissues, multiplex immunofluorescence imaging combined with scRNA-seq identified changes in innate immune cell populations (e.g., clusters of PD-L1+ macrophages) with potential roles in blocking T cell infiltration of the tumor lesion [97]. By comparing staining patterns in PMLs (e.g., AAH, AIS) and LUAD tissues, Wang et al. not only showed that immune infiltration increases in the LUAD relative to normal tissues, but also that heterogeneity and plasticity were a hallmark of immunomodulatory interactions since they varied greatly throughout tumor progression [40]. While many of these technologies are mostly used for validation purposes due to their limited number of simultaneously visible stains or probes (e.g., the use of digital spatial profiling platforms to detect biomarkers of good response to ICB in NSCLC [98]), few applications were successful in profiling a large number of targets (e.g., protein markers or transcripts), and thus cells, with some achieving this at near single-cell resolution. For instance, multiplexed hybridization-based techniques enabled studying the spatial distribution of around 2900 mouse lung cells including rare subsets, and subsequently, estimating how they interact with each other in vivo [99]. More recently, the use of spatial barcoding coupled with next generation sequencing, also known as “spatial transcriptomics”, has been increasingly useful in capturing transcriptomic data from defined locations on tissue sections. For example, spatial transcriptomic profiling has helped point towards new immune rich niches (e.g., IgA-producing plasma cells) in healthy airways, with potential roles in host defense mechanisms and in the context of respiratory infectious diseases [100]. The commercialization of user-friendly spatial omics platforms and their optimization to capture true single-cell resolution is anticipated to magnify the armamentarium of high definition spatial profiling of LUAD tissues at different stages of tumor progression, including rare, archived, and fixed samples [101].

Equally important, and prior to achieving a standardization of genomic and molecular profiling of PMLs (whether in the research or clinical setting), one cannot discount the need for routine, safe, and effective sampling of early lesions from patients (Figure 2). Aiding these advances are promising new robotics-assisted platforms that have been recently empowering physicians to reach deeper into the lung, including the ability to sample small pulmonary nodules at the periphery [102]. These devices are not only targeting lesions that were nearly impossible to reach manually, but they are also increasing the yield and sensitivity of tissue sampling as well as reducing the invasiveness of conventional bronchoscopy [102]. Parenthetically, delivery of ablative therapies to peripheral lung lesions using these advanced bronchoscopy tools is now also emerging as a highly viable option for a subset of patients [103]. Current and anticipated breakthroughs in bronchoscopic navigation and peripheral target lesion approximation will help circumvent the long-standing paucity in research accessible PMLs and rare lesions and will increase tissue accessibility for investigative purposes. Overall, these advances can have profound outcomes on our ability to profile early lesions at high resolution, and to study longitudinal cohorts of matched lung tissue samples harvested at different stages along the pathogenic continuum of lung cancer (Figure 2).

## 6. Summary and Perspectives

**Figure 3:**
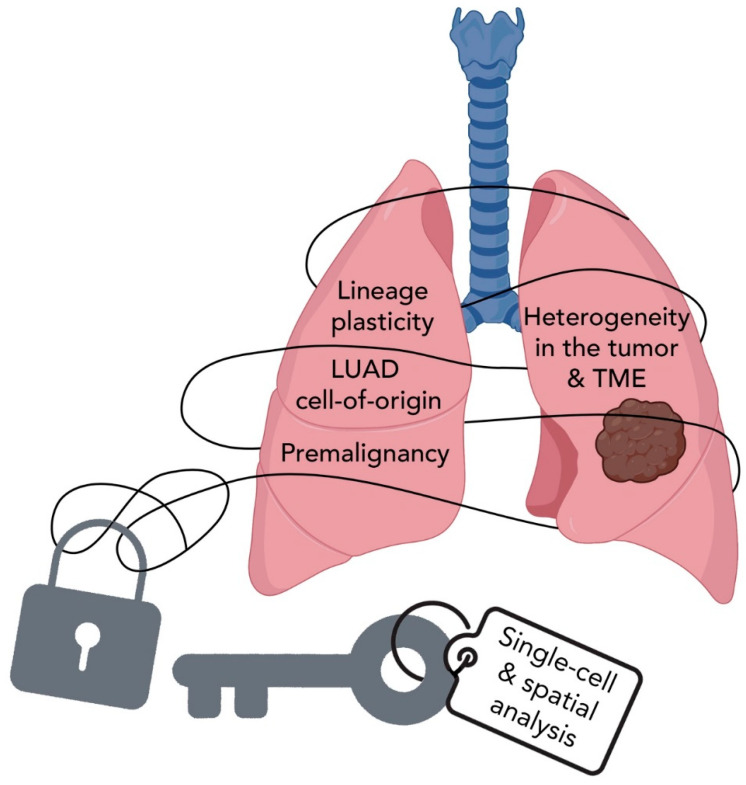
Single-cell RNA sequencing can help unravel the evolution of LUAD. Studying LUAD PMLs and tumor lesions at the single-cell and spatial levels has unlocked novel concepts and intriguing phenomena in the spatiotemporal evolution of the malignancy. These advances continue to elucidate our knowledge of cells-of-origin, lineage plasticity at early and late stages of tumor evolution, and the complexity of heterogeneity within the tumors and in their microenvironments. LUAD; lung adenocarcinoma, TME; tumor microenvironment.

The use of comprehensive molecular and genomic approaches to profile the transcriptomic landscape of a variety of LUAD lesions at the single-cell level has unraveled novel and intriguing concepts and phenomena in the spatiotemporal evolution of the malignancy. These advances have enhanced our knowledge of cells-of-origin, lineage plasticity at early and late stages of tumor evolution, and in response to various stress signals, the complexity of tumor heterogeneity, and the richness and roles of the TME landscape in lung pathogenesis and response to therapy (Figure 3).

The rewards from studying cells at a cell-by-cell level are being constantly amplified by ongoing advances in the technology of single-cell platforms which include but are not limited to: improving sequencing signal-to-noise ratio, expanding the scale of output data by capturing more cells per sample or achieving more sequencing reads per cell, standardizing techniques to better compare single-cell data across diverse studies and sample types, achieving true single-cell resolution in spatial transcriptomics, scaling and commercializing multi-omic single-cell platforms to simultaneously profile multiple analytes per single cell (e.g., RNA, DNA, copy number and single-nucleotide polymorphisms, chromatin states, and cell–surface protein expression), and finally, reducing sample preparation and sequencing costs.

Of note, a better understanding of LUAD development and improved opportunities for early detection can be strongly supported by decoding underlying mutational mechanisms at the single cell level (e.g., single-cell DNA-sequencing; scDNA-seq). For instance, by sequencing the genomes of single cells from normal lung tissues from healthy donors or high-risk smokers, we can have an improved understanding of the earliest molecular events that can push a normal cell down the path of neoplastic transformation. To achieve this, there have been a few tools that aim to circumvent the main hurdle in scDNA-seq, namely a high error rate in base calling. These surrogate methods include sequencing of clones or organoids cultured from single cells [104,105,106], or of monoclonal cells from micro-dissected tissues [107,108]. For instance, sequencing individual micro-dissected colonic crypts has been recently applied to study the genomic landscape of normal epithelial in patients with Lynch syndrome, a hereditary disease that predisposes patients to colon cancer [108]. Unfortunately, these methods are only applicable to a limited range of tissue types. While molecular barcoding, the standard tool for scRNA-seq, may be a solution to bypass this issue in scDNA-seq, the technology would necessitate very high sequencing depth, thereby making it less appealing for broader use compared to scRNA-seq [109]. With the rapid advancements in sequencing technologies and bioinformatics, we anticipate a widespread use of scDNA-seq in cancer studies in the near future, and perhaps even the possibility to assess both the genome and the transcriptome from the same single cell. In parallel, bioinformatics tools continue to improve in refining the scale and accuracy with which to infer cellular properties and behaviors, such as cell trajectories, crosstalk, and mutation status (e.g., somatic mutation calling from scRNA-seq data). Single-cell technologies for studying LUAD are now also benefiting from engineering advances that are creating new technical solutions to safely and accurately obtain rare, early, and peripheral lung lesions from high-risk patients. Taken together, the future of single-cell research will certainly continue to provide a more holistic view of LUAD initiation and progression across time and space. These advances promise new advances in clinical management of this morbid disease, be it by preventing tumor progression at early stages or by improving the prognosis of late-stage LUADs.
